# *Streptococcus suis* in Swedish grower pigs: occurrence, serotypes, and antimicrobial susceptibility

**DOI:** 10.1186/s13028-020-00533-3

**Published:** 2020-06-24

**Authors:** Anna Werinder, Anna Aspán, Annette Backhans, Marie Sjölund, Bengt Guss, Magdalena Jacobson

**Affiliations:** 1 Department of Clinical Sciences, Swedish University of Agricultural Sciences (SLU), Box 7054, 750 07 Uppsala, Sweden; 2 Department of Microbiology, National Veterinary Institute (SVA), 751 89 Uppsala, Sweden; 3 Department of Animal Health and Antimicrobial Strategies, National Veterinary Institute (SVA), 751 89 Uppsala, Sweden; 4 Department of Biomedical Science and Veterinary Public Health, Swedish University of Agricultural Sciences (SLU), Box 7036, 750 07 Uppsala, Sweden

**Keywords:** Antibiotic resistance, Bacteria, Environmental factors, Infectious diseases, Meningitis, Microbiology, Porcine, Streptococci, Swine, Zoonosis

## Abstract

**Background:**

*Streptococcus suis* is a major cause of meningitis, arthritis, and pneumonia in pigs worldwide, and an emerging pathogen in humans. In Sweden, *S. suis* has previously received little attention but has in recent years become increasingly recognized as affecting the pig production. The aim of the present study was to investigate the occurrence, serotypes and antimicrobial susceptibility of *S. suis* in Swedish grower pigs from herds with and without reported *S. suis* associated disease, as well as possible associations between *S. suis* associated disease and selected environmental and production factors. Swab samples were taken from the tonsils of clinically healthy 8–13-week-old grower pigs from ten case herds and ten control herds. Isolates were cultured, identified using MALDI–TOF MS, and serotyped using latex agglutination. The antimicrobial susceptibility of 188 isolates was tested using broth microdilution. Production data was gathered and environmental parameters were measured on the farms.

**Results:**

*Streptococcus suis* was isolated from 95% of the sampled pigs in both the case and the control herds. Serotypes 3, 4, 5, 7, 9, 10, 11, 15, 16, and 17–34 were detected, although a majority of the isolates (81.5%) were non-typeable. There was less diversity among the serotypes isolated from the case herds than among those from the control herds; four and nine different serotypes, respectively. Isolates resistant to penicillin (3.8%) were reported for the first time in Sweden. Tetracycline resistance was common (88.4%). No association was noted between the production and the environmental factors investigated, and the carriership of *S. suis*.

**Conclusions:**

The carriership of *S. suis* was found to be higher in clinically healthy Swedish pigs than previously estimated, and for the first time, the presence of Swedish isolates resistant to penicillin was reported. Many of the most commonly disease-associated serotypes, e.g. serotypes 2, 9, 3, and 7, were detected in healthy grower pigs although further studies are needed to investigate the virulence of these isolates.

## Background

*Streptococcus suis* is considered one of the most important pathogens affecting pig production worldwide and is also an emerging zoonotic agent in humans. In both humans and pigs, *S.* *suis* may cause meningitis, sepsis, arthritis, pneumonia, endocarditis, and acute death [1, 2]. Pigs carry the bacteria in the tonsils and on the nasal mucosa, as well as in the gastrointestinal and genital tracts [3, 4], and healthy carrier animals are thought to be present on most pig farms. Transmission of bacteria between pigs occurs mainly via the respiratory route [5], and from sows to piglets at birth [6].

Environmental factors such as higher outside temperature, excessive temperature fluctuations, and high air relative humidity have been associated with a higher proportion of *S. suis* carriership, as have production factors such as crowding and continuous production systems [7, 8]. Further, infection with porcine reproductive and respiratory syndrome (PRRS) virus may predispose pigs to secondary *S.* *suis*-infections [9].

Presumptive diagnosis in pigs is based on clinical signs and should be confirmed by necropsy and isolation of the pathogen. *S. suis* is a phenotypically and genetically diverse species with a complex taxonomy and may be challenging to accurately identify in the laboratory. Based on the antigenicity of the polysaccharide capsule, 35 serotypes were initially described, although sequencing of the 16S rRNA and *cpn60* genes has more recently indicated that six of these belong to other species [10–12]. The serotypes most commonly isolated from diseased pigs are 2, 9, 3, 1/2, and 7 [13], and individual pigs frequently carry more than one serotype [14, 15]. Relatively high levels of resistance to some antimicrobials (e.g. macrolides, lincosamides, tetracyclines, and sulphonamides) have been reported in many countries, while resistance to penicillins has generally been described as low [16].

Although the first Swedish reports of *S. suis*-infections in pigs and humans were published in the mid-1980s [17, 18], this pathogen has until recently received little attention. However, two more human cases were reported in 2014 [19, 20], and veterinarians in clinical practice have noticed an increase in pigs showing clinical signs indicative of *S.* *suis*-infection. Therefore, the aim of this study was to investigate the occurrence, serotypes and antimicrobial susceptibility of *S.* *suis* in Swedish grower pigs from farms with and without reported disease, as well as possible associations with selected environmental and production factors.

## Methods

The study was approved by the Ethics Committee for Animal Experimentation, Uppsala, Sweden (Dnr 5.8.18-15404).

### Study design

A case–control design was used. Herds were chosen based on the referrals of herd health veterinarians from the Swedish “Farm & Animal Health” service that covers 80% of the Swedish pig herds. The case group included ten pig herds where the health-service veterinarian had previously diagnosed *S.* *suis*-infections and clinical signs had been noted in several batches of weaned pigs during the year preceding the sampling. An equal number of herds where no such diagnoses had ever been made by the herd health veterinarians were used as controls. No selected herds opted out of participating. For the purposes of this study, clinical signs indicative of *S.* *suis*-infection were defined as grower pigs (from weaning to 30 kg body weight) circling or exhibiting seizures, lateral recumbency with paddling leg movements, or severe pneumonia in herds where *S. suis* pneumonia had previously been laboratory confirmed.

### Herds

The samples were collected during 2018 and 2019 from 20 farms located in the south and central parts of Sweden, in the counties with the highest pig density. The herds included piglet producing, farrow-to-finish, and gilt producing conventional herds, as well as piglet producing and farrow-to-finish organic herds, and piglet producing and farrow-to-finish sow pool satellites (i.e., a multi-site production system with a central unit for mating and pregnant sows, and several satellite herds where farrowing and piglet production takes place [21]). In accordance with the Swedish legislation [22], growth-promoting antibiotics or growth-promoting hormones were not used. Further, the Swedish pig production is declared free from PRRS virus, and an active surveillance programme is in place [23].

### Data collection

In each farm, environmental measurements were collected from the room housing the targeted pigs. Temperature and air relative humidity were measured at the height of approximately 1 m in the middle of the room using a Testo 625 thermohygrometer (Testo SE & Co. KGaA, Lenzkirch, Germany). The air velocity was measured at the height of approximately 0.1 m in 2–6 randomly distributed pens per room, adjacent to the solid and to the slatted floor areas (or, where applicable, adjacent to the deep straw bedding), using a Testo 405-V1 thermal anemometer (Testo SE & Co. KGaA). Carbon dioxide was measured at the height of approximately 1 m in the middle of the room and ammonia was measured at the height of approximately 0.1 m adjacent to the slatted floor area in 4–6 randomly distributed pens per room, (or, where applicable, adjacent to the deep straw bedding), using colorimetric detector tubes and a manual GV-100 air sampling pump (GASTEC Corporation, Kanagawa, Japan).

Information on management practices and production data covering 1 year before sampling was obtained from the farmers through interviews and from the farm management software PigVision (AgroVision B.V., Deventer, Netherlands). All data collection was performed by the first author. Mean values for the case and control group were compared using a two-tailed t-test, and P < 0.05 was considered significant. Statistical analysis was performed in R version 3.6.2 [24].

### Bacterial sampling

From each farm, samples were taken from ten clinically healthy grower pigs from the same batch, if possible from one pig per pen. The pigs were 8–13 weeks of age and had not been subjected to any treatment for at least 1 month before sampling. The pigs had all been weaned at between 4 and 6 weeks of age [25]. A sample was obtained from each pig’s palatine tonsils by opening the mouth using snares of braided nylon rope around the upper and lower jaws and rubbing an eSwab™ 480CE (Copan Diagnostics, Inc., Corona, CA, USA) on the tonsillar surface for 3 s. The swabs were immediately placed in tubes containing liquid Amies transport medium, transported to the laboratory at ambient temperature, and were processed for bacteriological analysis within 18 h of sampling.

### Bacterial isolation and identification

The swabs were streaked directly onto streptococcal selective colistin-oxolinic acid-blood agar (COBA) plates (National Veterinary Institute, Uppsala, Sweden), and incubated at 37 °C in 5% CO_2_ overnight. From each sample, 5–12 small, translucent colonies exhibiting α- or β-hemolysis [26] were subcultivated on 5% horse blood agar plates (National Veterinary Institute) and incubated at 37 °C in 5% CO_2_ overnight. Following incubation, matrix-assisted laser desorption ionization-time of flight mass spectrometry (MALDI-TOF MS) was used to identify the bacterial colonies to the species level. Material from 1 to 3 pure-cultured colonies was smeared directly onto a polished steel target (Bruker Daltonik GmbH, Bremen, Germany), covered with 1 μL of α-cyano-4-hydroxycinnamic acid (HCCA) matrix (Bruker Daltonik GmbH), prepared according to the manufacturer’s instructions, and allowed to dry at room temperature. MALDI-TOF MS analysis was performed using a Microflex LT System running version 4.1.60 of the BDAL database (Bruker Daltonik GmbH). A positive species identification was defined as a MALDI-TOF MS score ≥ 2.00 [27]. Isolates identified with low confidence (MALDI-TOF MS scores between 1.70 and 1.99) were re-tested using the direct transfer-formic acid method [28], where 1 μL of 70% formic acid (Sigma-Aldrich, Steinheim, Germany) was added to the bacteria on the target spot and allowed to air dry before matrix solution was added and analysis performed. A maximum of five confirmed *S. suis* isolates per pig were preserved at − 70 °C, and later one isolate per pig was randomly selected for serotyping and antimicrobial susceptibility testing.

### Serotyping by latex agglutination

Isolates were serotyped by latex agglutination [20, 29] using the commercially available Immulex™ *S. suis* kit (SSI Diagnostica A/S, Hillerød, Denmark) according to the manufacturer’s instructions. The test identifies serotypes 1 through 16 separately, and groups serotypes 17 through 34 together. Briefly, 1 μL of colony material, pure-cultured on 5% horse blood agar at 37 °C in 5% CO_2_ overnight, was suspended in 250 μL of sterile saline before mixing 10 μL of the suspension with 10 μL of latex reagent. Agglutination that occurred within 60 s was interpreted as a positive reaction.

### Antimicrobial susceptibility testing

Antimicrobial susceptibility testing was performed by broth microdilution using commercially available VetMIC GP-mo panels (National Veterinary Institute), according to the manufacturer’s instructions. Isolates were cultured on 5% horse blood agar plates and incubated at 37 °C in 5% CO_2_ overnight. Colonies were suspended in a 0.9% sterile saline solution to obtain an inoculum density of 5 × 10^5^ colony-forming units per mL (CFU/mL), which was added to cation-adjusted Mueller–Hinton broth (National Veterinary Institute) supplemented with 3% lysed horse blood (Håtunalab AB, Bro, Sweden). Each of the 96 wells of a VetMIC GP-mo plate was inoculated and incubated aerobically at 37 °C for 18–20 h. *S. suis* ATCC 43765 and *Streptococcus pneumoniae* ATCC 49619 were used as control strains and minimum inhibitory concentrations (MIC) were within the accepted quality control ranges. The MIC was recorded as the lowest concentration of an antimicrobial inhibiting visible bacterial growth. MICs of cefoxitin, cefalotin, ciprofloxacin, clindamycin, chloramphenicol, enrofloxacin, erythromycin, gentamycin, penicillin, tetracycline, trimethoprim, and trimethoprim/sulfamethoxazole were determined. The results were interpreted according to Clinical and Laboratory Standards Institute (CLSI) breakpoints for *S. suis* [30], where available.

## Results

### Herds

The case group consisted of seven farrow-to-finish herds and three piglet-producing herds. Out of the ten case herds, three were sow pool satellites from different sow pools. The control group consisted of eight farrow-to-finish herds, one gilt-producing, and one piglet-producing herd. Out of the ten control herds, two were specific pathogen-free (SPF) herds [31] and three were organic herds providing access to outside pastures or exercise yards for the pigs. All in-all out production was practiced consistently in all but one case and one control herd. The time for which the nursery pig barns were allowed to sit empty between batches varied between and within herds, in the case group from 0 to 21 days and in the control group from 0 to 7 days.

Clinical signs indicative of meningitis predominated in eight out of ten case herds, while two herds reported respiratory signs and acute deaths due to *S. suis.* All herds had recorded at least one case during the month preceding the sampling. At the interviews, three control herds reported that single pigs with clinical signs consistent with an *S.* *suis*-infection had been observed by the farmers on single occasions. These potential cases had however not been clinically confirmed by the herd health veterinarians.

There were no significant differences between case and control herds in the environmental parameters measured (Table 1). Taking the farm owners’ reports into account and reclassifying the three control herds reporting occasional cases as case herds, did not affect these results.

**Table 1:** Environmental parameters investigated in 20 Swedish pig herds with (case herds) and without (control herds) a history of *Streptococcus suis*-infections in grower pigs

Environmental parameters	Control herds (n = 9)^a^		Case herds (n = 10)		P value^b^
	Mean	SD	Mean	SD	
Temperature (°C)	20.4	3.42	19.5	3.95	0.59
Carbon dioxide (ppm)	1622	950	1550	695	0.85
Ammonia in pens, mean (ppm)	6.6	3.3	6.6	2.6	0.63
Air relative humidity (%)	55.9	17.5	58.4	12.4	0.73
Air velocity (solid floor or deep straw bedding), mean (m/s)	0.10	0.05	0.09	0.03	0.34
Air velocity (slatted floor), mean (m/s)^c^	0.14	0.08	0.07	0.03	0.10

^a^Biosecurity rules in one herd prohibited measuring equipment being brought in

^b^P values were calculated using Welch’s t-test. P < 0.05 was considered significant

^c^Herds using deep straw bedding not included, leaving control herds n = 6 and case herds n = 9

No significant differences were found between the case and control herds regarding the production data parameters (Table 2). However, reclassifying the three control herds reporting occasional cases as case herds resulted in the number of sows farrowing per batch being significantly lower (P = 0.01) in the control herds (n = 7; mean 22.7, SD 12.5) than in the case herds (n = 13; mean 40.1, SD 13.0).

**Table 2:** Production data from 20 Swedish pig herds with (case herds) and without (control herds) a history of *Streptococcus suis*-infections in grower pigs

Production parameters	Sweden 2017^a^	Control herds			Case herds			P value
	Mean	n	Mean	SD	n	Mean	SD	
Sows in production^b^	354	9	223.1	178.8	7	424.3	345.9	0.15
Sows farrowing per batch	n/a	10	28.7	14.2	10	39.3	14.8	0.12
Litters/sow/year^b^	2.24	6	2.12	0.27	6	2.20	0.06	0.49
Piglets born alive/litter	14.3	7	14.0	0.9	8	14.5	0.7	0.34
Pigs weaned/litter^c^	11.9	7	11.3	1.1	8	11.9	0.7	0.22
Pigs weaned/sow/year^b^	26.6	6	24.0	4.6	6	26.6	1.4	0.24
Age at weaning (days)	32.8	10	36.0	4.2	10	33.2	3.3	0.11
Gilt recruitment (%)	24.8	9	25.7	9.5	8	29.0	5.5	0.40
Farrowing interval (weeks)	n/a	10	3.4	1.7	10	2.5	1.1	0.18

All data parameters were not available for all herds (n = number of herds included)

^a^Mean values for Sweden according to the InterPIG report 2018 [49]

^b^Sow pools not included

^c^Nurse sows were used in one of the control herds and six of the case herds

The total number of sows in the sow pools ranged from 1000 to 1700, with between 40 and 55 sows farrowing in each batch at the sampled satellite farms.

### Bacterial sampling

*Streptococcus suis* was isolated from at least eight out of ten (mean 9.5, SD 0.7) of the sampled animals in each control herd, and from at least eight out of ten (mean 9.5, SD 0.7) of the sampled animals in each case herd. In total, *S. suis* was isolated from 95% (190 out of 200) of the sampled animals included in the study.

### Serotyping

**Fig. 1:**
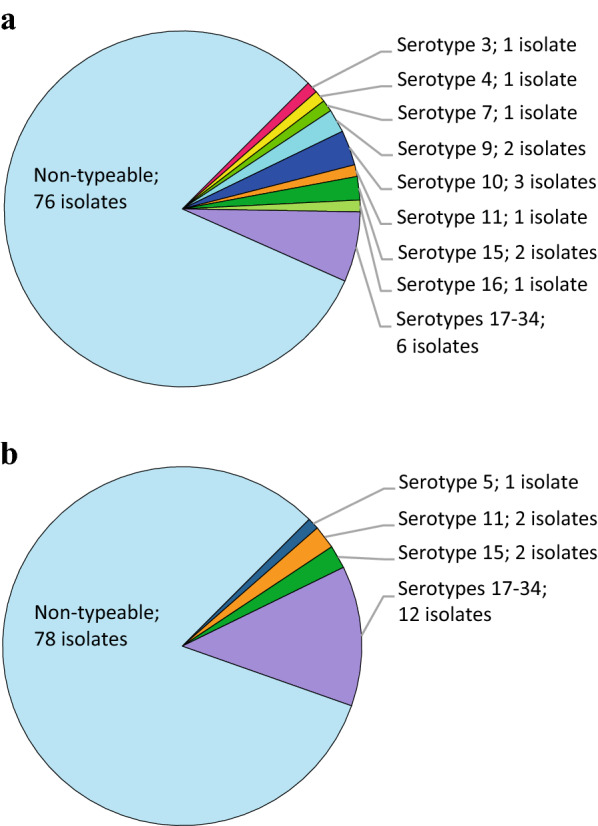
Serotypes of *Streptococcus suis* isolated from the tonsils of clinically healthy 8–13-week-old grower pigs (n = 189) in Sweden. One isolate per pig was tested with latex agglutination. **a** Isolates (n = 94) from ten control herds with no history of *S. suis*-infections. **b** Isolates (n = 95) from ten case herds with a history of *S. suis*-infections

Latex agglutination was performed on 189 out of 190 isolates (one isolate could not be re-cultivated for analysis). A majority of the isolates (81.5%, 154 out of 189) exhibited insufficient or no agglutination and were therefore serologically non-typeable. The percentage of non-typeable isolates did not differ (82.1% and 80.9%, respectively) between case and control herds. The distribution of serotypes is shown in Fig. 1.

Serotype 5 was detected only in one case herd, whereas serotypes 3, 4, 7, 9, 10, and 16 were detected only in the control herds. The isolates of serotypes 3, 4, 7, 10, and 16 were from one herd each, whereas serotype 9 was found in two herds. Serotypes 11, 15, and 17–34 were detected in both case and control herds.

Reclassification of the three previously mentioned control herds with occasional single cases of suspected *S. suis*-infections as case herds, resulted in one isolate from each of the serotypes 3, 7, 9, 15, and 16 being included in the case group.

### Antimicrobial susceptibility testing

Antimicrobial susceptibility testing (Table 3) was performed on 188 of the 190 isolates obtained from clinically healthy pigs (two isolates could not be re-cultivated for analysis). Susceptibility to penicillin was determined for 184 isolates (four isolates were not able to grow in the presence of the citric acid-containing buffer used for penicillin).

**Table 3:**
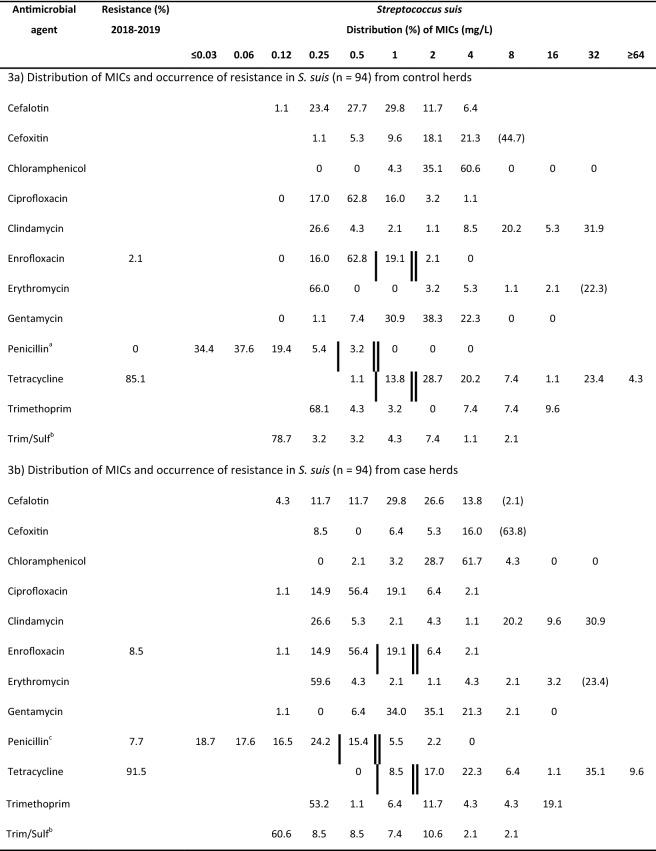
Antimicrobial susceptibility of 188 *Streptococcus suis* isolates from clinically healthy Swedish grower pigs from ten control herds without a history of *Streptococcus suis*-infections and ten case herds with a history of *Streptococcus suis*-infections in grower pigs

Isolates from (3a) case herds and (3b) control herds were obtained during 2018–2019 and tested using broth microdilution. Species-specific breakpoints according to CLSI 2018 [30] are indicated with single vertical lines (sensitive) and double vertical lines (resistant). Values for the lowest concentration tested indicate MICs lower than or equal to the lowest concentration within the range. Parentheses indicate isolates with MICs above the dilution range included in the test panel

^a^n = 93 in the case of penicillin

^b^Concentration for trimethoprim given, tested in combination with sulfamethoxazole in a concentration ratio of 1:20

^c^n = 91 in the case of penicillin

Several isolates (9.2%, 17 out of 184) were classified as intermediate, and 3.8% (7 out of 184) were resistant to penicillin. A majority of these not susceptible isolates (87.5%, 21 out of 24), including all of the resistant isolates, originated from the case herds. The percentage of isolates not susceptible to enrofloxacin was 27.7% in the case group and 21.3% in the control group while the percentages of isolates not susceptible to tetracycline were similar in the two groups, at 100% and 98.9%, respectively.

## Discussion

Compared to the limited historical data available [32, 33], *S. suis* was isolated from a higher percentage than expected of clinically healthy Swedish grower pigs in both case and control herds. There was no difference in the percentage of carriers between the groups, and interestingly, the conventional herds did not differ in this respect from the closed SPF-herds or the organic herds with lower stocking density and outdoor access.

In contrast to previous studies on Swedish isolates [33, 34] the present study reports the occurrence of reduced susceptibility and resistance to penicillin. This is notable since penicillin resistance in *S. suis* is generally reported to be uncommon [16], and since Sweden has very low sales of antimicrobials for the use in food-producing animals [35]. In Sweden, benzylpenicillin is the most common antibiotic sold for the use in pig production, and in 2014 the consumption of antibiotics for pigs consisted of 75% products for injection, of which 60% were products containing benzylpenicillin [36]. A majority of the not susceptible isolates originated from case herds, which may be because of potentially higher use of antibiotic substances in these herds.

Despite the low Swedish sales of tetracyclines, as compared to sales in other European countries [35, 37], the present study demonstrates a very high percentage of isolates, 100% of isolates from the case group and 98.9% isolates from the control group, to be “not susceptible” i.e. intermediate or resistant, to tetracycline. Out of all the tested isolates, 88.4% were resistant to tetracycline, which is markedly higher than the 7.7% reported in 1998 [33], and the 82.0% reported in 2017 [34].

Using the CLSI breakpoints defined for enrofloxacin [30], 73.4% of the isolates were classified as susceptible. It is of note that the breakpoint has been determined for a dose of 7.5 mg/kg [38], and it is not valid for lower dosages such as the one authorized in Sweden (5 mg/kg). Thus, the clinical usefulness of enrofloxacin for the treatment of *S. suis* infections is questionable.

All of the isolates investigated originated from clinically healthy pigs, and several previous studies have also shown that resistance to several classes of antibiotics is more common in non-clinical isolates than in isolates from diseased pigs [39–42]. It must also be considered that differences in the sampling strategies, susceptibility testing methodologies, and interpretive criteria applied, complicate comparisons of antimicrobial susceptibility data from different studies. Additionally, the lack of species-specific veterinary clinical breakpoints for several classes of antibiotics hampers the clinical interpretation of the results.

Environmental factors such as higher outside temperature, temperature fluctuations, and a relative humidity of > 70%, have previously been associated with a higher carriership of *S. suis* in clinically healthy pigs [7, 8]. In the present study, the relative humidity and temperature in the room were measured once before bacterial sampling commenced, and no association was found between these parameters and the carriership of *S. suis*. Temperature logging, outside and in the pig barn, over a longer period of time and in a greater number of herds may be considered in the future when assessing possible disease-triggering factors.

In addition, none of the investigated production factors differed significantly between case and control herds. However, if the three herds that had experienced single clinical cases of presumptive *S. suis*-infections were reclassified as case herds, the number of sows farrowing per batch was significantly higher in this group. Since the number of routes of transmission increases with an increasing number of individuals this might indicate that the group size is of importance. However, production data was not available for some herds, and care should be taken when interpreting these results.

The herds in the study were included in the case or control group solely based on their herd health veterinarian’s assessment, as judged by the clinical picture and laboratory results. The few participating SPF- and organic farms were all found in the control group, which could indicate that clinical problems with *S. suis* are less common problem in these herds. However this interesting observation needs further investigations.

Several of the most commonly disease-associated *S. suis* serotypes, e.g. serotypes 9, 3, and 7, were detected in clinically healthy pigs this study. *S. suis* is often considered part of the normal flora of the tonsils, and although certain serotypes are more often associated with disease, virulence can also vary within serotypes [43]. The diversity among the serotypes was lower in the case herds than in the control herds; four and nine serotypes, respectively. It is, however, difficult to draw any conclusions based on the present results. A majority (81.5%, 154 out of 189) of the investigated isolates were non-typeable using the latex agglutination method. This result may be due to poor sensitivity of the method used but is in accordance with several previous studies that, depending on the method used, have demonstrated up to 67% of isolates from clinically healthy pigs to be non-typeable [40, 43–45]. These isolates may be non-encapsulated or possess novel capsular polysaccharide loci [46]. The serotype group 17–34 encompasses six serotypes (20, 22, 26, 32, 33, and 34) that have been reclassified as *S.* *parasuis*, *S.* *orisratti*, and *S.* *ruminantium* [11, 12, 47]. Thus, further studies are needed to assess the virulence of these isolates. Other serotyping methods, e.g. in silico serotyping based on whole-genome sequencing, may be useful to further investigate the serotypes present in Sweden.

The perceived low incidence of clinical disease in Sweden may be due to low virulence of the strains present, or it may be related to other factors such as a high weaning age, a legislated minimum space allowance for growing pigs that is higher than the EU minimum [25, 48], or to the generally high health standard of pig herds, e.g. the freedom from PRRS virus [23]. There is however also the possibility that *S.* *suis*-infection may be underreported or misdiagnosed, and that the pathogen might be a more common cause of disease than previously acknowledged. Further, the possibility of the MALDI–TOF MS method generating false-positive results cannot be excluded.

## Conclusion

This study shows *S. suis* to be more common in Swedish pig herds than previously estimated, and for the first time reports the presence of Swedish isolates resistant to penicillin. Several of the most commonly disease-associated serotypes were isolated from clinically healthy grower pigs, although a large number of isolates were serologically non-typeable using latex agglutination. Further studies are needed to investigate the serotypes and virulence of these isolates. No association was noted between the environmental factors investigated and the carriership of *S. suis* in clinically healthy grower pigs.

## Data Availability

The datasets used and/or analysed during the current study are available from the corresponding author on reasonable request.

## Abbreviations

CFU: Colony-forming units
CLSI: Clinical & Laboratory Standards Institute
COBA: Colistin-oxolinic acid-blood agar
HCCA: α-Cyano-4-hydroxycinnamic acid
MALDI-TOF MS: Matrix-assisted laser desorption ionization-time of flight mass spectrometry
MIC: Minimum inhibitory concentration
PRRS: Porcine reproductive and respiratory syndrome
SPF: Specific pathogen free, i.e. free from infections with Mycoplasma hyopneumoniae, Actinobacillus pleuropneumoniae, toxigenic Pasteurella multocida, Brachyspira hyodysenteriae, swine influenza virus, and Sarcoptes scabiei. SPF production is also free from the following pathogens which are not present in Sweden: African swine fever virus, Aujeszky’s disease virus, Brucella species, classical swine fever virus, foot and mouth disease virus, Japanese encephalitis virus, porcine epidemic diarrhoea virus, porcine reproductive and respiratory syndrome virus, rabies virus, swine vesicular disease virus, and transmissible gastroenteritis virus.
